# Tumor-derived exosomal HMGB1 fosters hepatocellular carcinoma immune evasion by promoting TIM-1^+^ regulatory B cell expansion

**DOI:** 10.1186/s40425-018-0451-6

**Published:** 2018-12-10

**Authors:** Linsen Ye, Qi Zhang, Yusheng Cheng, Xiaolong Chen, Guoying Wang, Mengchen Shi, Tong Zhang, Yingjiao Cao, Hang Pan, Liting Zhang, Genshu Wang, Yinan Deng, Yang Yang, Guihua Chen

**Affiliations:** 1 Department of Hepatic Surgery and Liver Transplantation Center, The Third Affiliated Hospital of Sun Yat-sen University, Guangzhou, China; 2 Guangdong Provincial Key Laboratory of Liver Disease Research, Guangzhou, China; 3 Cell-gene Therapy Translational Medicine Research Center, The Third Affiliated Hospital of Sun Yat-sen University, Guangzhou, China; 4 Institute of Human Virology, Zhongshan School of Medicine, Sun Yat-sen university, Guangzhou, China; 5 Department of Pathology, The Third Affiliated Hospital of Sun Yat-sen University, Guangzhou, China

## Abstract

**Background:**

Regulatory B (Breg) cells represent one of the B cell subsets that infiltrate solid tumors and exhibit distinct phenotypes in different tumor microenvironments. However, the phenotype, function and clinical relevance of Breg cells in human hepatocellular carcinoma (HCC) are presently unknown.

**Methods:**

Flow cytometry analyses were performed to determine the levels, phenotypes and functions of TIM-1^+^Breg cells in samples from 51 patients with HCC. Kaplan-Meier plots for overall survival and disease-free survival were generated using the log-rank test. TIM-1^+^Breg cells and CD8^+^ T cells were isolated, stimulated and/or cultured in vitro for functional assays. Exosomes and B cells were isolated and cultured in vitro for TIM-1^+^Breg cell expansion assays.

**Results:**

Patients with HCC showed a significantly higher TIM-1^+^Breg cell infiltration in their tumor tissue compared with the paired peritumoral tissue. The infiltrating TIM-1^+^Breg cells showed a CD5^high^CD24^−^CD27^−/+^CD38^+/high^ phenotype, expressed high levels of the immunosuppressive cytokine IL-10 and exhibited strong suppressive activity against CD8^+^ T cells. B cells activated by tumor-derived exosomes strongly expressed TIM-1 protein and were equipped with suppressive activity against CD8^+^ T cells similar to TIM-1^+^Breg cells isolated from HCC tumor tissue. Moreover, the accumulation of TIM-1^+^Breg cells in tumors was associated with advanced disease stage, predicted early recurrence in HCC and reduced HCC patient survival. Exosome-derived HMGB1 activated B cells and promoted TIM-1^+^Breg cell expansion via the Toll like receptor (TLR) 2/4 and mitogen-activated protein kinase (MAPK) signaling pathways.

**Conclusions:**

Our results illuminate a novel mechanism of TIM-1^+^Breg cell-mediated immune escape in HCC and provide functional evidence for the use of these novel exosomal HMGB1-TLR2/4-MAPK pathways to prevent and to treat this immune tolerance feature of HCC.

**Electronic supplementary material:**

The online version of this article (10.1186/s40425-018-0451-6) contains supplementary material, which is available to authorized users.

## Introduction

Humoral immunity, in addition to cellular immunity, has recently been reported to play a key role in tumor progression [1, 2]. As the central component of humoral immunity, B cells function in immunoglobulin (Ig) production, antigen presentation, and proinflammatory cytokine secretion. Regulatory B (Breg) cells, a subset of B cells, have been confirmed to regulate immune responses through a variety of ways [3, 4]. Recent studies have revealed the existence of protumorigenic Breg cells in tumor tissue [5–8]. However, those Breg cells have showed different phenotypes and functions.

T cell Ig and mucin domain (TIM)-1, a transmembrane glycoprotein, has been identified as one of three members of the human TIM family of genes that regulate immune responses [9, 10]. TIM-1 was first identified to play a costimulatory role in the activation of CD4^+^ T cells and dendritic cells; in TIM-1 exerts an important effect by regulating cellular function [9, 11–13]. However, recent studies have confirmed that TIM-1 is mainly expressed by B cells and that TIM-1 acts as a marker for Breg cells [14]. TIM-1^+^Breg cells appear to include the highest proportion of IL-10-producing B cells, are 8–20-fold enriched for IL-10 expression compared with all other B cell subsets, and comprise over 70% of all IL-10 producing-B cells [15]. Their regulatory function and relationship with systemic autoimmune disease or transplantation have been characterized [15–18]. However, the biological function of TIM-1^+^Breg cells in the human tumor microenvironment has not been illustrated.

Exosomes are endosome-derived small intraluminal vesicles that are characterized by a size range from 30 to 200 nm in diameter and contain a variety of biological substances, including miRNAs, circRNAs, proteins, lipids, and soluble factors, that are transferred to target cells to play roles as important mediators in cell-to-cell communication. Exosomes derived from tumor cells have been reported to regulate the phenotypes and functions of immune cells [19–23]. Recently, High mobility group box 1 (HMGB1) was demonstrated to be expressed on tumor-derived exosomal membranes and packaged in human amnion epithelial cell-derived exosomes (AECD-exosomes) [24] [25]. HMGB1 is an evolutionarily conserved DNA-binding nuclear protein that has been determined to be a damage molecular pattern (DAMP) protein involved in several disease states, including cancer, arthritis and sepsis [26]. Tumor cells can release HMGB1 into the local microenvironment, where HMGB1 binds with high affinity to several receptors, such as toll like receptor-2 (TLR-2), TLR-4, TLR-9 and advanced glycation end products (RAGE), which can lead to tumor cell survival, expansion and metastasis [27]. However, whether HMGB1 expressed on tumor-derived exosomes can induce protumorigenic Breg production is not well known.

## Materials and methods

### Patients samples and cell lines

Human liver and HCC samples from resected HCC were obtained from the Third Affiliated Hospital of Sun Yat-sen University. None of the patients had received anticancer therapy before surgical resection, and patients with concurrent HIV, other cancer or autoimmune disease were excluded. Paired tumor and peritumoral liver tissue samples and fresh samples of blood from 51 HCC patients who underwent curative resection between September 2016 and August 2017 were used to isolate immune cells (Additional file 1: Table S1). Among the samples, those from 20 HCC patients treated between December 2016 and March 2017 were used to analyze gene expression and perform immunoblotting. The tissue samples were used for immunohistochemistry (IHC) or immunofluorescence (IF) at the same hospital where the patients underwent surgical resection between September 2011 and December 2012 (Additional file 2: Table S2). Clinical stages were classified according to the guidelines of the International Union against Cancer. Patient consent was obtained from each patient, and the protocol was approved by the Review Board of the Third Affiliated Hospital of Sun Yat-sen University. HuH7, HepG2, Hep3B, LM3, and LO2 cells were obtained from ATCC.

### Isolation of immune cells from the peripheral blood and tissue samples

Peripheral blood mononuclear cells (PBMCs) were isolated using a standard Ficoll procedure. Tissue-infiltrating leukocytes were dissociated from tissue specimens and collected as described previously [28]. Specimens were cut into small pieces and dissociated using RPMI 1640 medium (Gibco) supplemented with 0.05% collagenase IV (Sigma-Aldrich), 0.002% DNase I (Roche), and 20% FBS (Gibco) at 37 °C for 1 h. Dissociated cells were filtered through a 150-mm mesh and separated by Ficoll centrifugation (Axis-Shield), and the mononuclear cells were washed with HBSS and resuspended in RPMI 1640 medium supplemented with 10% FBS.

### Flow cytometry

Immunophenotyping was performed on a BD flow cytometer (BD LSR2) and the identification of exosomes was performed on a BD flow cytometer (Accuri C6) following the standard manufacturer’s protocols. The results were analyzed using FlowJo 10.0 software. The immune cells were stained with the fluorochrome-conjugated antibodies used as listed in Additional file 3: Table S3. Detailed staining protocol were previously described [28]. For the intracellular staining of B cells, brefeldin A (10 mg/ml; Sigma-Aldrich) was added during the last 4 h of stimulation. The cells were washed with PBS and stained with monoclonal antibodies specific for CD19, CD27, CD38, CD5, CD24 or CD8 for 30 min at 4 °C. After the cells were washed in PBS, they were fixed and permeabilized with a fixation/permeabilization solution (eBioscience, San Diego, CA, USA) for 20 min at room temperature. Then, the cells were washed again and stained with monoclonal antibodies against IL-10, TNF-α and IFN-γ for 30 min at room temperature.

### Immunohistochemistry and immunofluorescence

The tissues were fixed in 4% paraformaldehyde (Sigma-Aldrich) and then cut into 4-um sections, which were processed for immunohistochemistry as previously described [28]. Then, the sections were incubated with a primary antibody against human TIM-1 (Sigma-Aldrich). The staining was detected by a Dako-Cytomation Envision Horseradish Peroxidase System (DakoCytomation). Two independent observers who were blinded to the clinical outcomes evaluated the immunohistochemical variables.

For the immunofluorescence analysis, paraffin-embedded human HCC sections were stained with mouse anti-CD20 (Abcam) plus rabbit anti TIM-4 (Abcam) antibodies, followed by FITC-conjugated anti-rabbit IgG plus CY3-conjugated anti-mouse IgG antibodies (Abcam). Positive cells were detected by confocal microscopy (LSM 510, Carl Zeiss; ZEN 2010 software). All antibodies are listed in Additional file 4: Table S4.

### Isolation of human CD19^+^ B or CD8^+^ T cells from the peripheral blood

Peripheral blood mononuclear cells were isolated from healthy donor blood samples by Ficoll centrifugation (Axis-Shield). CD19^+^ B or CD8^+^ T cells (more than 95% CD19^+^ or CD8^+^) were purified from peripheral blood mononuclear cells using a MACS CD19^+^ B or CD8^+^ T cell Isolation Kit (Miltenyi Biotech).

### Elisa

For the collection of tumor-infiltrating TIM-1^−^ and TIM-1^+^ B cells, tumor–infiltrating leukocytes were dissociated from tissue specimens as described above. Then, tumor-infiltrating leukocytes were stained with the fluorochrome-conjugated antibodies anti-CD19-BV421 and anti-TIM-1-PE. TIM-1^−^ and TIM-1^+^B cells were harvested by FACS sorting. To measure the level of the cytokine IL-10, 1 × 10^5^ TIM-1- and TIM-1^+^B cells were plated in 96-well plates and cultured for 72 h. Then, the culture supernatants were collected for ELISA analysis. The levels of human IL-10 were determined using human ELISA kits (R&D Systems) according to the manufacturer’s instructions.

### Gene expression analysis

A quantitative PCR (qPCR) assay was performed on a total of 20 mRNA samples isolated from the HCC tumor tissue and peritumoral liver tissue samples as described previously [29]. Real-time PCR for mRNA detection was performed using SYBR Green PCR Master Mix (Roche). Data were normalized to the reference gene GAPDH. The primers used were as follows: HMGB1 primers: FW’ ACATCCAAAATCTTGATCAGTTA, RV’ AGGACAGACTTTCAAAATGTTT and GAPDH primers: FW’ GGGAAGCTTGTCATCAATGG, RV’ CATCGCCCCACTTGATTTTG.

### Western blot analysis and Wes automated simple western assay

Whole-cell proteins were extracted and analyzed using the procedure for a normal western blot as previously described [28]. The Wes simple western system is an automated capillary electrophoresis with immunodetection approach that eliminates the blotting steps and improves accuracy, reproducibility and quantification compared with normal western blotting [30]. The identification of exosomes was performed using a Wes simple western instrument (Wes, Protein Simple, Santa Clara, CA). Wes Master Kits were used following the standard manufacturer’s protocols. The results were imaged and analyzed using Compass software. The antibodies were used as listed in Additional file 4: Table S4.

### Exosome isolation from cell lines

Exosomes were isolated by differential centrifugation of conditioned media collected from various cell lines, including HuH7, HepG2, Hep3B, LM3 and LO2 cells. All culture medium contained polymyxin B (20 μg/ml; Sigma-Aldrich) to eliminate endotoxin contamination. The cells were grown in their respective conditioned media to 70 to 80% confluency. The medium was then replaced with medium containing FBS depleted of microparticles by differential centrifugation (18 h at 100,000×*g*). After a 48 h incubation, the conditioned media were initially cleared of dead cells/cellular debris with sequential spins at 300×*g* for 10 min at 4 °C. The resulting supernatants were then spun at 11,200×*g* for 32 min at 4 °C. The supernatant was filtered through a 0.22 μm screen to remove large vesicles. Then, the supernatant was centrifuged again at 100,000×*g* for 120 min at 4 °C. The final exosome pellet was resuspended in 1× PBS filtered through a 0.22 μm screen.

### Transmission electron microscopy (TEM)

Exosomes suspended in PBS filtered through a 0.22 μm screen were layered on copper grids with 0.125% Formvar in chloroform and stained with 1% uranyl acetate in ddH_2_O. The exosomes were then placed on a transmission electron microscopy grid, and images were acquired using a Hitachi H-7650 transmission electron microscope.

### NanoTracker analysis of exosomes

Exosome fractions isolated from the cell lines were diluted to meet the appropriate concentration analyzed on a ZETASIZER Nano series-Nano-ZS. The videos were merged and analyzed using the NanoSight® software program. The results show the particle size distribution vs. intensity (percent).

### TIM-1^+^ B cell induction in vitro

CD19^+^ B cells (2 × 10^5^ cells/well) isolated from healthy blood were left unprocessed or exposed to CpG ODN (InvivoGen, 2 μg/mL), recombinant Human HMGB1 (R&D Systems, 10 μg/mL), or exosomes from LO2, HuH7, HepG2, Hep3B and LM3 cells (2–3 μg in 50 μL PBS) prepared for 3 days or the indicated time. The cells were harvested for western blotting or stained with fluorochrome-conjugated antibodies and then analyzed by FACS. In some experiments, CD19^+^ B cells were pretreated with 2 μg/mL CpG ODN, 10 μg/ml anti-HMGB1, 20 μg/ml blocking antibody against TLR-2 or TLR-4 (eBioscience) or a specific inhibitor of the p38 (SB 203580,20 μM), Erk (U 0126,20 μM), or Jnk (SP 600125,5 μM) signal (Sigma-Aldrich) and subsequently exposed to the indicated stimuli.

### CFSE-based CD8^+^ T cell proliferation assay and cytokine production assays

CD19^+^ B cells (2 × 10^5^ cells/well) in a 96-well plate were harvested after exposure to CpG ODN plus recombinant human HMGB1 or exosomes for 3 days. Next, the cells were collected, washed with PBS and centrifuged at 400×*g* for 5 min at 4 °C. CD8^+^ T cells were harvested from the same healthy person at the same time and activated with IL-2 (150 IU/ml, PeproTech) for 3 days. CD8^+^ T cells were labeled with 1.5 μM CFSE (Thermo Fisher Scientific) in 0.1% BSA in PBS for 5 min at 37 °C and quenched with cold PBS. Then, CFSE-labeled CD8^+^ T cells were seeded at 10^5^ cells per well in a 96-well plate in 100 μl of RPMI 1640 medium containing 10% FBS. TIM-1^+^ B cells add to the CD8^+^ T cells at a ratio of 1:1. Next, the CD8^+^ T cells were activated by the addition of 2 μl anti-CD3 and 5 μl anti-CD28 beads (eBioscience) per well for 3 days. Subsequently, CD8^+^ T cell proliferation and TNF-α and IFN-γ expression was measured by flow cytometry.

### Statistical analysis

The results are expressed as the mean ± SEM. The statistical significance of differences between groups was analyzed by the log-rank test or Student’s t test. Correlations between two parameters were assessed by Pearson’s correlation analysis. A multivariate analysis of the prognostic factors for the overall survival curve and disease-free survival curve was performed using the Cox proportional hazards model and log-rank test. The cumulative survival time was calculated using the Kaplan-Meier method. All data were analyzed using two-tailed tests, and *P* < 0.05 was considered the standard of statistical significance. *P < 0.05, **P < 0.01, ***P < 0.001 and ****P < 0.0001.

## Results

### High infiltration of TIM-1^+^ B cells is correlated with advanced disease stage and poor survival in patients with HCC

**Fig. 1:**
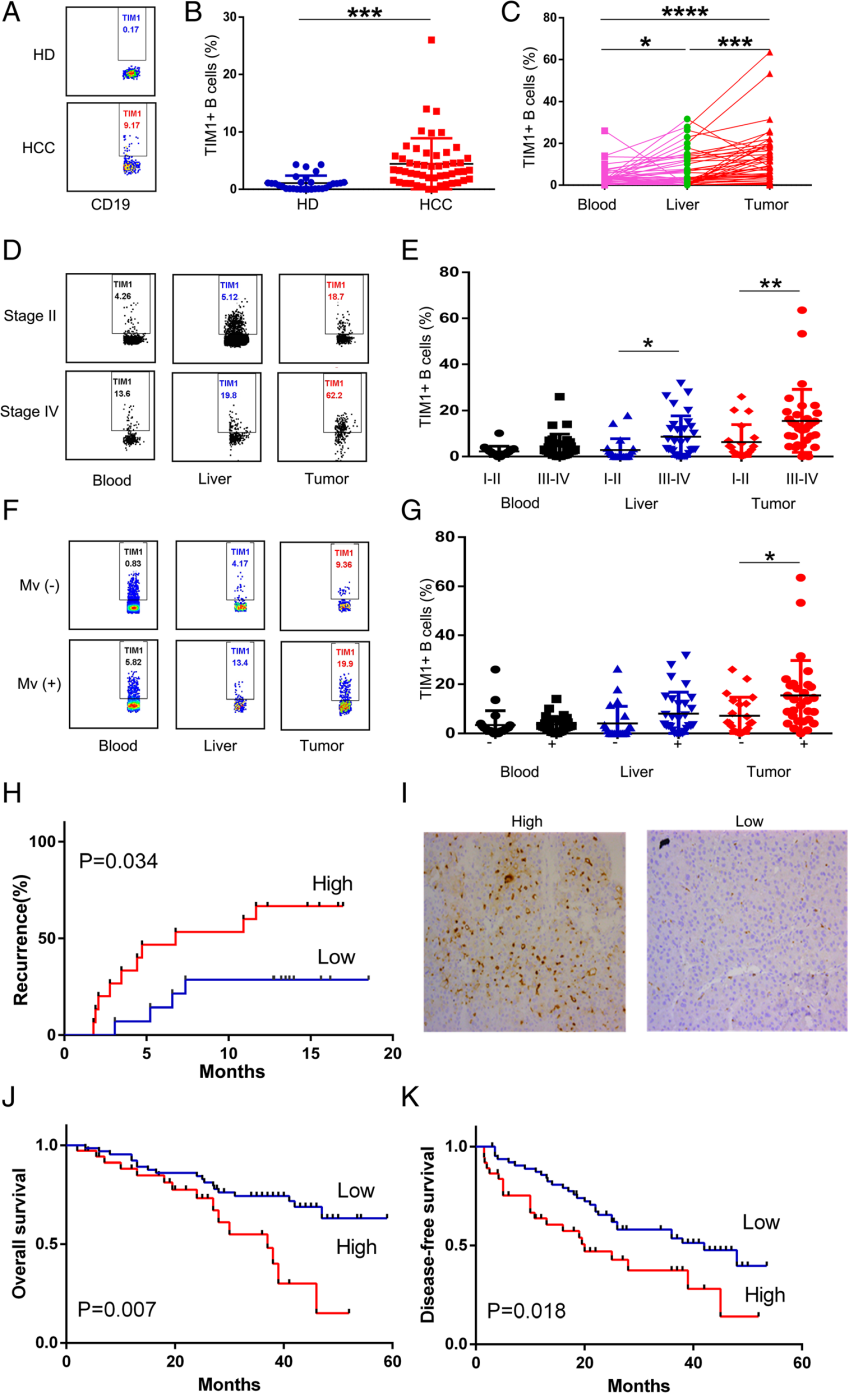
Strong infiltration of TIM-1^+^B cells is correlated with advanced disease stage and poor survival in patients with HCC. **a-b** TIM-1 expression on CD19^+^ B cells isolated from PBMCs from HCC patients (*n* = 51) and healthy donors (*n* = 30) was determined by flow cytometry. **a** One representative experiment is shown. **B** The data are represented as the mean ± s.e.m. **C** TIM-1^+^B cells from tumor tissue were compared to those from paired PBMC samples and peritumoral liver samples (n = 51). **d-g** The associations of tumor-infiltrating TIM-1^+^B cells with patient TNM staging (*n* = 20 for stage I and II, *n* = 31 for stage III and IV) and microvascular invasion (*n* = 22 for positive, *n* = 29 for negative) are shown. **d** and **f** One representative experiment is shown. **h** Patients were divided into two groups (Low/High) according to the median value of the tumor TIM-1^+^ B cell percentages shown in C; statistical comparisons were performed with the log-rank test. **i** Representative immunohistochemical staining of TIM-1^+^ cells in HCC samples (*n* = 101) from the patients who were divided into two groups (low, ≤8 cells (*n = 53)*; high,>8 cells (*n = 48*)) according to the median value of TIM-1 expression is shown. Cumulative OS and DFS times were calculated by the Kaplan–Meier method and analyzed by the log-rank test. *P < 0.05, **P < 0.01, ***P < 0.001 and ****P < 0.0001

We used flow cytometry to analyze the TIM-1 expression of B cells from 30 normal blood samples and 51 HCC specimens (Additional file 1: Table S1) comprising blood samples and paired peritumor liver and tumor tissue samples. TIM-1 was expressed on more circulating B cells in HCC patients than healthy donors (Fig. 1a, and b). The percentage of TIM-1^+^B cells in the HCC patients was significantly increased in the tumor compared to the blood and peritumor liver (Fig. 1c). Our results showed that the percentage of TIM-1^+^B cells in lung cancer patients was significantly increased in the tumor compared to the blood and peritumor lung (Additional file 5: Figure S1), which was similar to the HCC results. Importantly, the proportion of TIM-1^+^B cells in the tumor tissue was positively correlated with patient TNM stage (Fig. 1d, and e), microvascular invasion (Fig. 1f, and g) and early recurrence (Fig. 1h and Additional file 6: Table S5).

Based on our observation that high accumulations of infiltrating TIM-1^+^B cells are correlated with advanced disease stage, we subsequently examined the relationship between TIM-1 expression and the survival of patients with HCC (Fig. 1i). According to the median value of TIM-1 density, the HCC patients who underwent curative resection and had recorded follow-up data were divided into two groups (*n* = 101, Additional file 2: Table S2). Indeed, obviously negative associations were observed between TIM-1^+^ cell density and disease-free survival (n = 101, *p* = 0.018, Fig. 1j) or overall survival (n = 101, *p* = 0.007, Fig. 1k). Both the univariate analysis and multivariate analysis revealed that the TIM-1 expression level in the tumor tissue was an independent prognostic factor for DFS and OS (Additional file 7: Table S6).

### TIM-1^+^B cells are regulatory B cells that exhibit a unique phenotype and function

**Fig. 2:**
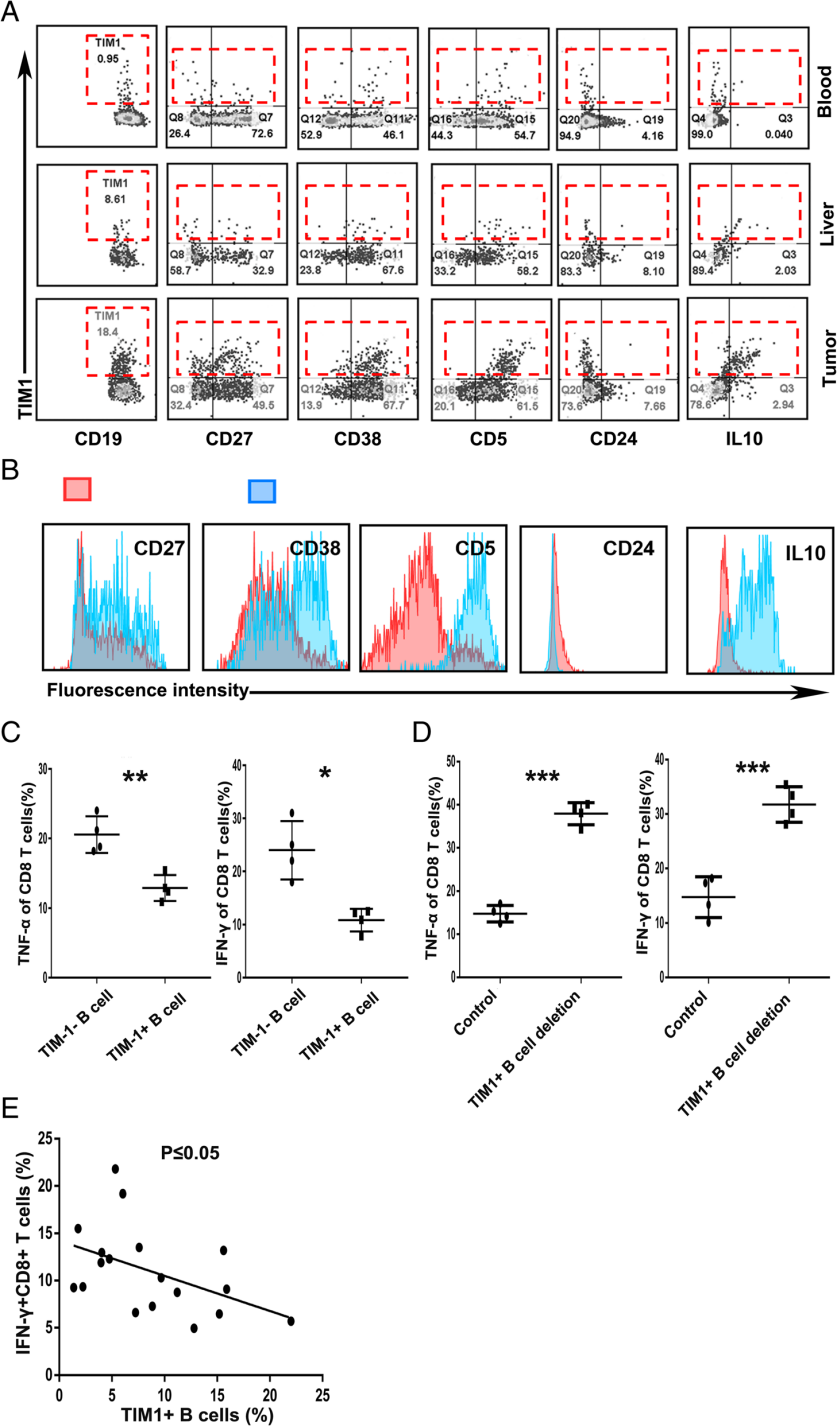
TIM-1^+^B cells are a regulatory B cell subset that exhibits a unique phenotype and function. **a** Flow cytometry analysis of the phenotypic characteristics of the TIM-1^+^B cells from the paired blood, liver tissue and HCC tissue samples (n = 10). **b** Analysis of the representative markers expressed by tumor-infiltrating TIM-1^+^ and TIM-1^−^B cells. **c** Flow cytometry analysis of the TNF-α and IFN-γ production of the tumor-infiltrating CD8^+^ effector T cells cocultured with tumor-infiltrating TIM-1^+^ B cells or TIM-1^−^ B cells. **d** Flow cytometry analysis of the TNF-α and IFN-γ production of CD8^+^ effector T cells in the tumor-infiltrating lymphocytes (TILs) and TILs without TIM-1^+^B cells groups. **c-d** The data are represented as the mean ± s.e.m. of three independent experiments. **e** Associations between tumor-infiltrating IFN-γ^+^CD8^+^ T cells and tumor-infiltrating TIM-1^+^B cells (*n* = 9). *P < 0.05, **P < 0.01, and ***P < 0.001

Since the accumulation of TIM-1^+^B cells was negatively correlated with DFS and OS in HCC patients, we used flow cytometry to investigate the phenotypic and functional characteristics of TIM-1^+^B cells from the blood, peritumor liver tissue, and tumor tissue from individual patients. As is well known, conventional peripheral IL-10-producing Breg cells are defined as CD5^+^CD24^high^CD27^+^CD38^high^ B cells. Interestingly, compared with the TIM-1^+^B cells from the paired blood and peritumor liver samples, tumor-infiltrating TIM-1^+^B cells exhibited a CD5^high^CD24^−^CD27^−/+^CD38^+/high^ phenotype, which is different from the conventional peripheral Breg phenotype (Fig. 2a). Consistently, the tumor-infiltrating TIM-1^+^B cells showed increases in the expression levels of CD5, CD27 and CD38 compared to the TIM-1^−^B cells (Fig. 2b). Several studies have reported that TIM-1 is an inclusive surface marker for IL-10-producing Breg cells [14–16, 31]. Indeed, our results showed that IL-10 was mainly expressed by TIM-1^+^B cells rather than TIM-1^−^B cells (Fig. 2b). Moreover, tumor tissue-derived TIM-1^+^B cells were potent suppressors of CD8^+^ T cell effector functions, as the TNF-α and IFN-γ production of CD8^+^ effector T cells was significantly decreased after coculture with TIM-1^+^ B cells or without depleting TIM-1^+^B cells (Fig. 2c, and d and Additional file 8: Figure S2). In addition, we further determined the relationship between TIM-1^+^B cells and CD8^+^ T cells in 18 HCC patients, and the results demonstrated that the frequency of CD8^+^ effector T cells negatively correlated with the frequency of TIM-1^+^B cells (Fig. 2e).

**Fig. 3:**
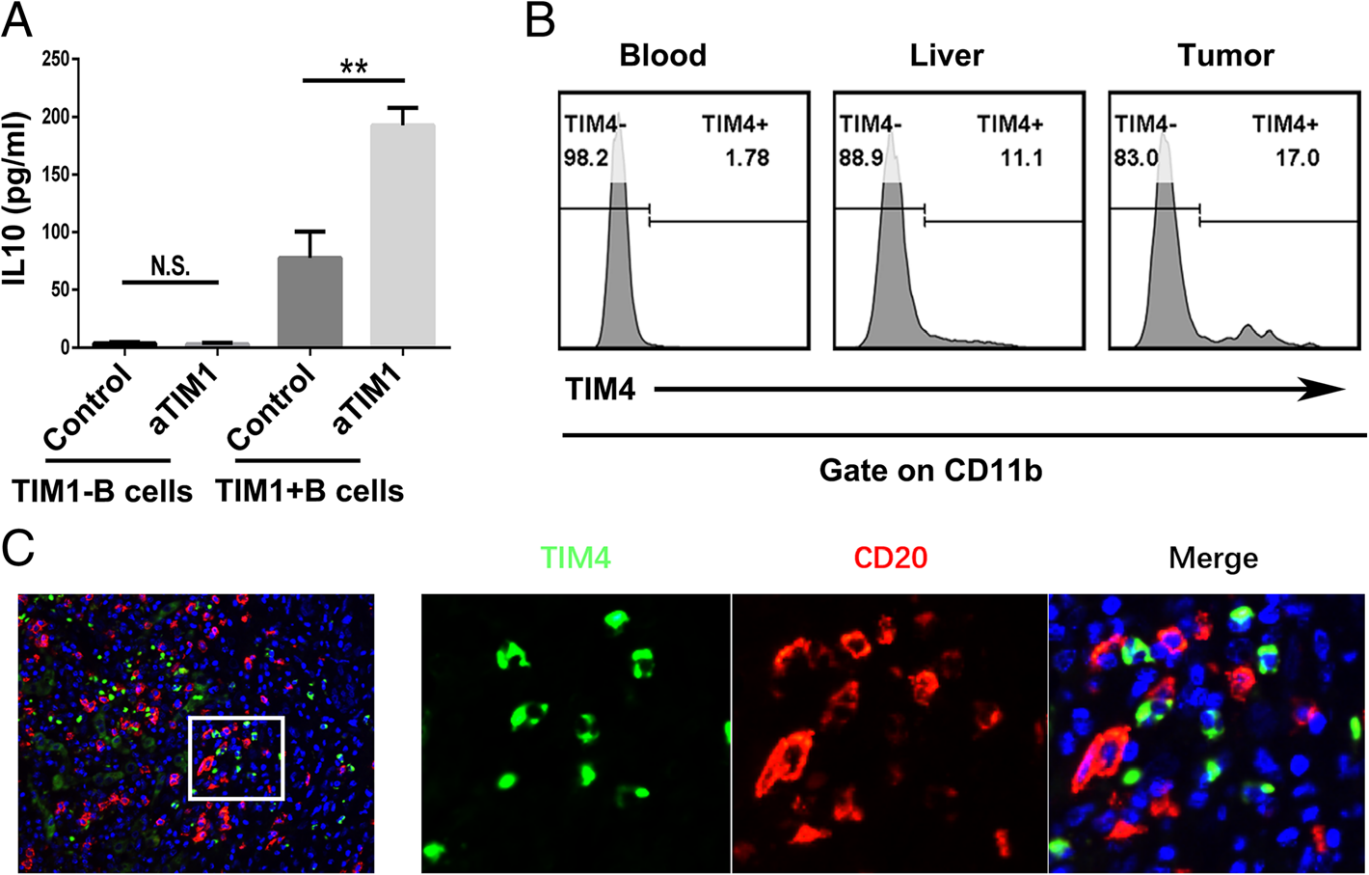
TIM-1^+^B cells produce IL-10 when TIM-1 interacts with its ligand. **a** Tumor-infiltrating TIM-1^−^ and TIM-1^+^ B cells (1 × 10^5^ cells/well) were purified by flow sorting and cultured in the presence of an anti-TIM-1 antibody (2 μg/ml) or PBS as control in vitro for 72 h. The supernatants from the cultures were collected, and the production of IL-10 was determined by ELISA (n = 3). **b** The flow cytometry analysis of TIM-4 expression on CD11b^+^ myeloid cells from the paired tumor tissue, liver tissue and blood samples (*n* = 14) is shown. **c** Representative immunofluorescence images of CD20 (red), TIM-4 (green) and nuclear staining with DAPI (blue) in HCC tissue are shown. **P < 0.01

As TIM-1 has been reported to be a potent regulator of T cell effector responses in both auto- and alloimmunity [12, 16, 17], we investigated the role of TIM-1 signaling in TIM-1^+^B cell function. Interestingly, an agonistic antibody against TIM-1 triggered TIM-1^+^B cells to produce IL-10 but showed no effect on the IL-10 production of TIM-1^−^B cells (Fig. 3a). Additionally, TIM-4, the natural ligand for TIM-1, was mainly expressed by CD11b^+^ myeloid cells (Fig. 3b), and the confocal microscopy results showed that TIM-4^+^ myeloid cells and B cells were located in the same region (Fig. 3c), which suggests that myeloid cells could promote TIM-1^+^B cells to produce IL-10 through TIM-1/TIM-4 signaling.

### Tumor-derived exosomes promoted TIM-1^+^B cell expansion via HMGB1

**Fig. 4:**
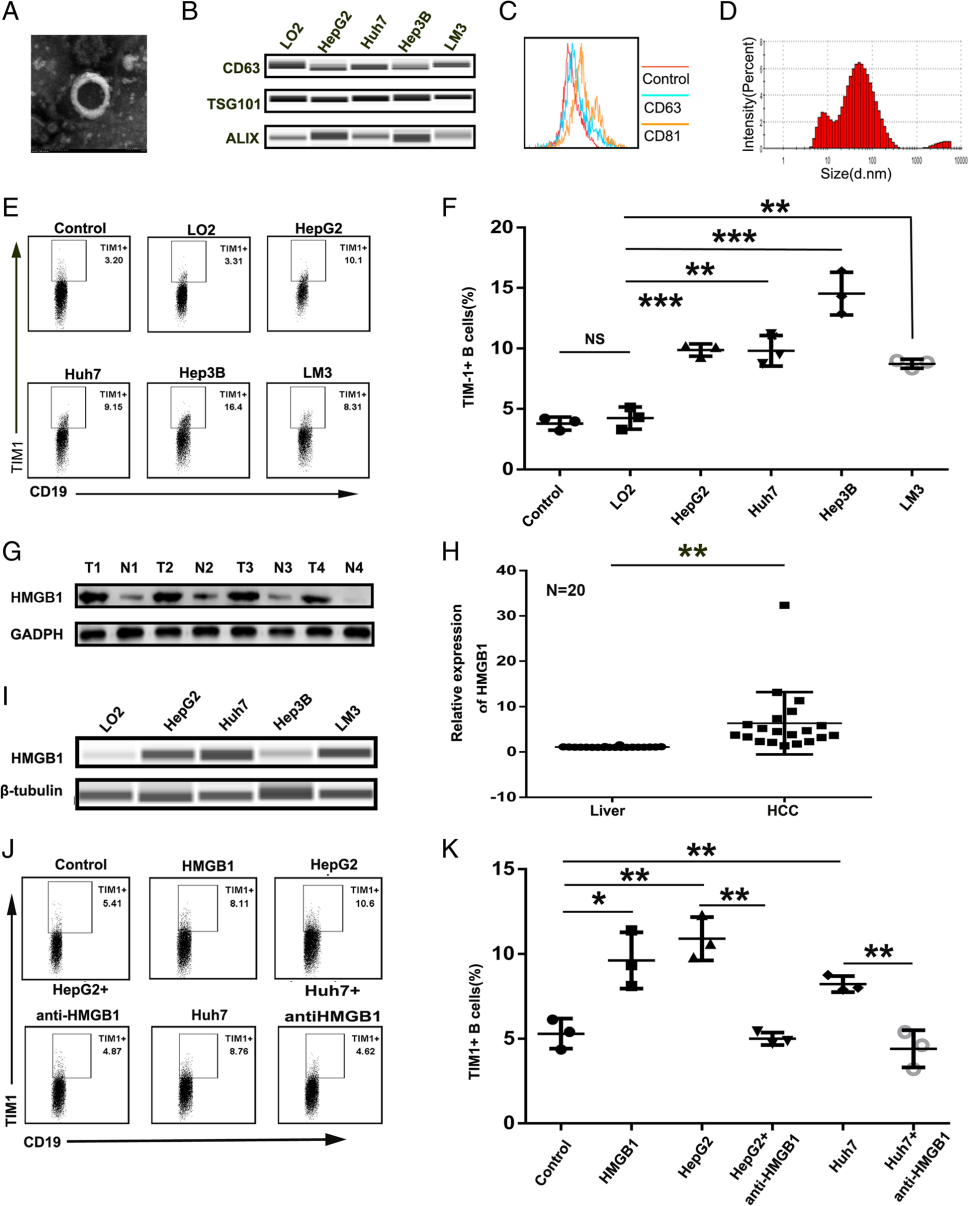
Tumor-derived exosomes promoted TIM-1^+^B cell expansion via HMGB1. Isolated exosomes were characterized by electron microscopy (**a**), western blotting (**b**), flow cytometry (**c**) and NanoTracker analysis (**d**). (**e**) After 3 days, CD19^+^ B cells (2 × 10^5^ cells/well) from healthy donor PBMCs were cultured in the presence or absence of exosomes (derived from cultures of LO2, HepG2, Huh7, Hep3B or LM3 cells) plus 2 μg/ml CpG ODN and then analyzed by flow cytometry to assess the frequency of TIM-1^+^B cells. **f** The dot plot represents the average percentages of TIM-1^+^B cells after culturing with or without exosomes (n = 3). **g-h** HMGB1 protein levels were measured using western blot analysis (**g**) and qPCR (**h**) in paired human HCC samples and their nontumor counterparts (N, nontumor tissue; T, tumor tissue; n = 20). **i** The HMGB1 level in exosomes (derived from LO2, HepG2, Huh7, Hep3B or LM3 cells) was measured by Wes automated simple western assay. **j** On day 3, CD19^+^ B cells (2 × 10^5^ cells/well) purified from healthy donor PBMCs were treated with HMGB1 (10 μg/ml) or exosomes (derived from LO2, HepG2 or Huh7 cells) in the presence or absence of an anti-HMGB1 antibody (10 μg/ml) and were analyzed by flow cytometry to assess the frequency of TIM-1^+^ B cells. **k** Dot plots represent the average percentages of TIM-1^+^ B cells after culturing with HMGB1 or exosomes in the presence or absence of the anti-HMGB1 antibody (n = 3). *P < 0.05, **P < 0.01, *** *P* < 0.001

Exosomes have been shown to play an important in the communication between immune cells and cancer cells by transferring important cellular cargo [22, 23, 32]. We therefore determined the effect of tumor-derived exosomes (TDEs) on TIM-1^+^B cell expansion. Exosomes were isolated from cell lines (LO2, HepG2, Huh7, Hep3B, and LM3 cells) by differential centrifugation and identified by electron microscopy, western blotting, flow cytometry and NanoTracker analysis (Fig. 4a, b, c, and d). Tumor-derived exosomes showed a strong potential to induce the substantial development of TIM-1^+^B cells from healthy blood B cells, whereas hepatocyte-derived exosomes failed to induce the generation of TIM-1^+^B cells (Fig. 4e, and f).

Due to HMGB1 playing a key role in tumor progression, we analyzed the amount of HMGB1 in 20 HCC tissue samples and their paired peritumor liver samples by qPCR (Fig. 4h) and also analyzed four paired samples by western blotting (Fig. 4g). Consistent with a previous report [26], our results showed that the expression of HMGB1 was significantly higher in tumor tissue than in peritumor liver tissue. The expression of HMGB1 was much higher in HCC-derived exosomes than LO2-derived exosomes (Fig. 4i). HMGB1 was found to be expressed on the exosomal membrane (Additional file 9: Figure S3), and the major source of HMGB1 in the cultures was not the active or passive release of HMGB1 by B cells (Additional file 10: Figure S4). To determine whether HMGB1 could facilitate the expansion of TIM-1^+^ B cells, we added HMGB1 or a neutralizing antibody against HMGB1 to the culture system and found that HMGB1 alone could facilitate the production of TIM-1^+^B cells, while the neutralizing antibody against HMGB1 could reverse the promotive effect of tumor-derived exosomes on the development of TIM-1^+^B cells (Fig. 4j, and k).

### Tumor-derived exosomes trigger B cells to exhibit strong suppressive activity against CD8^+^ T cells via HMGB1

**Fig. 5:**
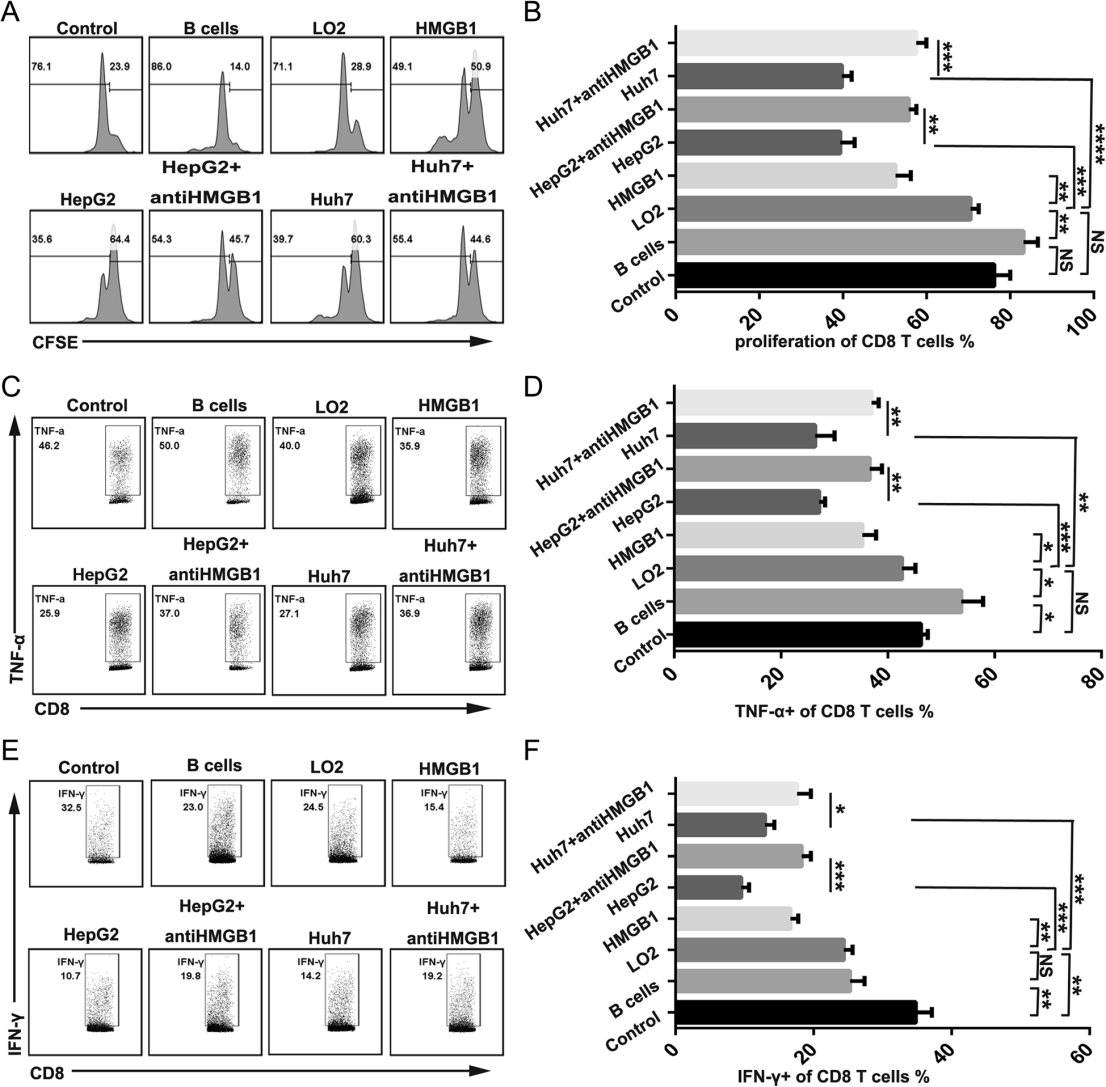
Tumor-derived exosomes trigger B cells to exhibit strong suppressive activity against CD8^+^ T cells via HMGB1. CD19^+^ B cells (2 × 10^5^ cells/well) were cultured with or without HMGB1 or TDEs (tumor-derived exosomes) in the presence or absence of an anti-HMGB1 antibody plus 2 μg/ml CpG ODN in 96-well plates. After 3 days, B cells were collected and cocultured with autologous CD8^+^ T cells labeled with 5,6-carboxyfluorescein (CFSE) in the presence of anti-CD3/CD28 mAb bead stimulation, and then CD8^+^ T cell proliferation (**a-b**)and TNF-α(**c-d**) and IFN-γ (**e-f**) production were analyzed by flow cytometry after 3 days. **a, c and e** One representative experiment of three is shown. **b, d and f** The data are represented as the mean ± s.e.m. of three independent experiments. *P < 0.05, **P < 0.01, *** P < 0.001

Next, we investigated whether TDEs or HMGB1-induced TIM-1^+^B cells could inhibit CD8^+^ T cell proliferation and function. For this aim, CD19^+^ B cells and CD8^+^ T cells were purified from the same human PBMC sample. As described above, TIM-1^+^B cells induced by TDEs or HMGB1 were coculture with CD8^+^ T cells that were stimulated using anti-CD3/CD28 mAb beads. Compared with the CD8^+^ T cells alone or noninduced B cells cultured with the CD8^+^ T cells groups, the groups with TDE- or HMGB1-induced TIM-1^+^B cells cocultured with CD8^+^ T cells demonstrated direct suppression of CD8^+^ T cell proliferation and TNF-α and IFN-γ production by the TIM-1^+^B cells, whereas the anti-HMGB1 antibody group showed significantly decreased suppression of CD8^+^ T cell proliferation and function by TIM-1^+^B cells (Fig. 5a, b, c, d, e, and f).

### Tumor-derived exosomal HMGB1 induced TIM-1^+^B cell expansion through the TLR-MAPK pathway

**Fig. 6:**
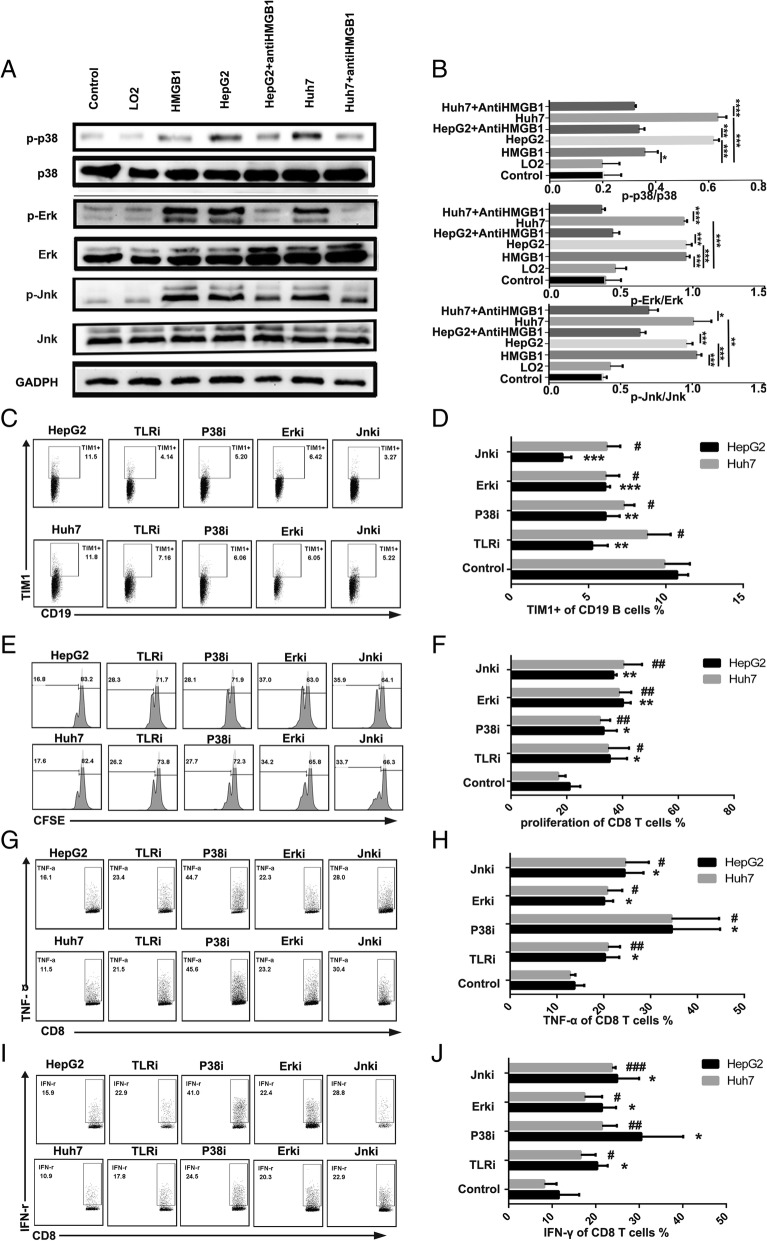
Tumor-derived exosomal HMGB1 induced TIM-1^+^Breg cell expansion through the TLR2/4-MAPK pathway. **a** B cells were cultured with HMGB1 and TDEs in the presence or absence of an anti-HMGB1 antibody, and the phosphorylation of P38, JNK and ERK in B cells was determined by western blotting. **b-c** CD19^+^ B cells (2 × 10^5^ cells/well) purified from healthy donor PBMCs were treated with exosomes (derived from LO2, HepG2 or Huh7 cells) in the presence or absence of TLR2/4, P38, JNK and ERK inhibitors in 96-well plates. After 3 days, the B cells were collected and analyzed by flow cytometry to assess the frequency of TIM-1^+^ B cells. **b** One representative experiment of three is shown. **c** The data are represented as the mean ± s.e.m. of three independent experiments. **d-i** CD19^+^ B cells were cultured with TDEs as described previously. TDE-triggered B cells were collected and cocultured with autologous CD8^+^ T cells labeled with CFSE in the presence of anti-CD3/CD28 mAb bead stimulation. After 3 days, CD8^+^ T cell proliferation (**d-e**) and TNF-α (**f-g**) and IFN-γ (**h-i**) production were analyzed by flow cytometry. **d, f and h** One representative experiment of three is shown. **e, g and i** The data are represented as the mean ± s.e.m. of three independent experiments. *P < 0.05, **P < 0.01, *** *P* < 0.001, ^#^*P* < 0.05, ^##^*P* < 0.01, ^###^ *P* < 0.001

HMGB1 binds with high affinity to several receptors, including the receptor for advanced glycation end products (RAGE) as well as Toll like receptor (TLR)-2, − 4, and − 9 [26]. Our results showed that blocking TLR-2 or TLR-4 significantly downregulated the frequency of TIM-1^+^B cells compared with the control treatment, while blocking TLR-9 and RAGE did not affect the frequency of TIM-1^+^B cells (Additional file 11: Figure S5). A recent study [8] demonstrated that TLR signaling participates in PD-1^high^ B cell induction through the mitogen-activated protein kinase (MAPK) pathway. To further confirm the mechanisms involved in the induction of TIM-1^+^B cells by HMGB1, we examined the activation kinetics of MAPK, which is downstream of TLR2/4 signaling. Activation of the MAPK pathways was selectively enhanced in B cells stimulated with TDEs or HMGB1 compared with the other stimulations, and the activation of the MAPK pathways was inhibited with anti-HMGB1 antibody treatment (Fig. 6a and b).

To determine the role of the TLR2/4-MAPK pathway in the TIM-1^+^B cell expansion mediated by tumor-derived exosomal HMGB1, a TLR2/4 blocking reagent and MAPK inhibitors were added to the culture system. Our results showed that blocking TLR2/4 significantly downregulated the frequency of TIM-1^+^B cells and impaired the suppressive effects of TDE-induced B cells on CD8^+^ T cell proliferation and TNF-α and IFN-γ production (Fig. 6c, d, e, f, g, h and i). MAPK inhibitors also effectively downregulated the frequency of TIM-1^+^B cells and impaired the suppressive effects of TDE-induced B cells on CD8^+^ T cell proliferation and TNF-α and IFN-γ production (Fig. 6c, d, e, f, g, h and i).

## Discussion

Understanding the cellular and molecular underpinnings of HCC-associated immune modulation is a prerequisite for the development of immunotherapy-based treatment approaches for this deadly malignancy. Here, we identified a previously unrecognized protumorigenic TIM-1^+^Breg cell subset and applied multiple analysis approaches to analyze the phenotype, biological function, mechanisms of induction, and clinical relevance of these cells in the HCC microenvironment. Our results showed that TIM-1^+^Breg cells exhibited a novel phenotype and function that were different from those of the conventional peripheral Breg cells [33, 34]. Moreover, tumor-derived exosomes induced B cells to become TIM-1^+^Breg cells through the HMGB1-TLR2/4-MAPK pathway. Additionally, strong infiltration of TIM-1^+^B cells was correlated with advanced disease stage and poor survival in patients with HCC.

Breg cells have been recently identified as a major contributor to the pathogenesis of autoimmune diseases and cancer. Breg cells have been reported to be enriched in patients with cancer and to be associated with advance disease stage and poor prognosis [8, 35, 36]. However, Breg cells derived from different tumor microenvironments exhibit distinct phenotypes and functions. Here, we demonstrated that in the context of HCC, TIM-1^+^Breg cells, an unrecognized protumorigenic B cell subset, exhibited a CD5^high^CD24^−^CD27^−/+^CD38^+/high^ phenotype. Importantly, TIM-1 was not only a surface marker but also a functional marker because triggering this molecule with an agonistic antibody induced TIM-1^+^ Breg cells to produce IL-10. Additionally, we identified that the TIM-1 ligand TIM-4 was mainly expressed on myeloid cells, which were determined to come into contact with B cells in the HCC microenvironment, suggesting that myeloid cells could induce TIM-1^+^ Breg cells to produce IL-10 through TIM-1/TIM-4 signaling. Thus, these results showed that TIM-1^+^Breg cells in HCC are different from conventional peripheral Breg cells.

In mice, TIM-1 has been reported to be an importance receptor for the induction and function of Breg cells, which preserve transplant immune tolerance and prevent autoimmunity [14, 31]. Many studies have showed that the BCR, CD40L and IL-21 pathways are required for IL-10-producing Breg cell induction and development [37]. However, recent studies have found that TIM-1 ligation could induce IL-10-producing Breg cells, and TIM-1 signaling was also required for the suppression of proinflammatory cytokine production by Breg cells. Moreover, some reports have indicated that the interaction between TIM-1 and its ligand plays a crucial role in Th2 polarization [38]. TIM-4 is the natural ligand of TIM-1 and is expressed on DCs [38, 39]. In our investigation, we found that TIM-4 was mainly expressed on myeloid cells, which induced TIM-1^+^Breg cells to produce IL-10 through TIM-1/TIM-4 signaling.

TIM-1^+^Breg cells have been shown to be involved in autoimmune disease and transplantation tolerance. However, the underlying mechanism contributing to the accumulation of TIM-1^+^Breg cells remains to be elucidated. With the emergence of exosomes as central players in HCC pathogenesis and the expanding list of reported exosome-mediated immunomodulatory effects [21–23, 40], we hypothesized that HCC-derived exosomes could play a role in regulating immune cells during tumor progression. HMGB1 was recently reported to be expressed on tumor-derived exosomes [24] and packaged in human AECD exosomes [25]. HMGB1 has been determined to play both protumorigenic and antitumorigenic roles. HMGB1 can act as an antitumorigenesis protein when it is located in the nucleus, where it stabilizes the chromosomes and maintains telomere length. On the other hand, extracellular HMGB1 is recognized as a prototypical DAMP, which has the protumorigenic role of promoting tumor progression [41]. In our study, HMGB1 was highly expressed on tumor-derived exosomes but not on hepatocyte-derived exosomes. The treatment of B cells with exosomes from HCC resulted in an increase in TIM-1^+^ Breg cell abundance, which was dependent on exosome-associated HMGB1. The major source of HMGB1 in the cultures was the tumor-derived exosomal membrane, not the active or passive release of HMGB1 from B cells (Additional file 10: Figure S4). Moreover, HMGB1 derived from tumor-derived exosomes was particularly potent for inducing TIM-1^+^Breg cell expansion, which promoted HCC progression by impairing the function of CD8^+^ effector T cells. Thus, our study revealed a new mechanism of HMGB1-mediated HCC progression.

The mechanism of tumor cell survival, expansion and metastasis is binding to TLR-2, TLR-4, TLR-9 and RAGE, which mediates HMGB1-dependent activation [27]. However, the PI3K/AKT, MAPK, and NFκB pathways are all downstream of TLR signaling [42]. A recent study [8] demonstrated that TLR signaling participates in PD-1^high^ B cell induction through the MAPK pathway. Moreover, TLR signaling has been recognized to promote antigen presenting cell maturation and proinflammatory cytokine production [43]. In our study, by blocking the activity of TLR-2, TLR-4, TLR-9 and RAGE independently, we showed that the frequency of TIM-1+ Breg cells was downregulated only in the TLR-2 and TLR-4 blockade groups (Additional file 11: Fig. S5). Recent studies have demonstrated that the activation of TLR-dependent signaling and HMGB1-RAGE can stimulate autophagy [44]. However, we found no change in the levels of autophagy-related molecules during TIM-1^+^ Breg cell induction (Additional file 12: Figure S6). The TLR2/4-MAPK pathway was activated during TIM-1^+^ Breg cell expansion, and targeting these signaling molecules impaired HMGB1-mediated TIM-1^+^Breg cell expansion and the suppressive function of TIM-1^+^Breg cells against CD8+ T cells, which suggest that HCC promotes the accumulation of TIM-1^+^Breg cells through the TLR2/4-MAPK pathway. Thus, our study highlights a novel pathway whereby HCC cells are able to release exosomes with the potential to promote the accumulation of TIM-1^+^Breg cells.

Based on our data, we propose a model describing the role of TIM-1^+^Breg cells in HCC progression (Additional file 13: Figure S7). First, HCC cells release exosomes with the potential to promote the accumulation of TIM-1^+^Breg cells through the HMGB1-TLR2/4-MAPK pathway. Second, TIM-1^+^Breg cells create an immunosuppressive microenvironment by secreting IL-10 and impairing CD8^+^ T cell functions, which provides favorable conditions for HCC progression. Third, myeloid cells further strengthen the immunosuppressive function of TIM-1^+^Breg cells through TIM-1/TIM-4 signaling. In the future, therapeutics aimed at interfering with these pathological TIM-1^+^Breg cells and the HMGB1-TLR2/4-MAPK immunosuppressive pathway may be developed to provide novel strategies for HCC treatment.

## Additional files

Additional file 1: **Table S1.** Characteristics of the study population (*N* = 51. (DOCX 15 kb)

Additional file 2: **Table S2.** Characteristics of the study population (*N* = 101). (DOCX 15 kb)

Additional file 3: **Table S3.** Flow cytometry panels for analysis of immune cells. (DOCX 17 kb)

Additional file 4: **Table S4.** The antibodies used in our experiments. (DOCX 17 kb)

Additional file 5: **Figure S1.** TIM-1+ B cells strongly infiltrated lung carcinoma tissue. (A) The TIM-1^+^B cells in the tumor tissue were compared to those of the paired PBMC and peritumoral lung samples (*n* = 3). (B) The data are represented as the mean ± s.e.m. *P < 0.05. (TIF 173 kb)

Additional file 6: **Table S5.** Univariate and Multivariate Analysis of Prognostic Factors for recurrence-free survival and Overall Survival (*N* = 29). (DOCX 18 kb)

Additional file 7: **Table S6.** Univariate and Multivariate Analysis of Prognostic Factors for recurrence-free survival and Overall Survival (N = 101). (DOCX 19 kb)

Additional file 8: **Figure S2.** TIM-1^+^Breg cells are a regulatory B cell subset that promoted HCC progression by impairing the function of CD8^+^ effector T cells. (A) Flow cytometry analysis of the TNF-α and IFN-γ production of tumor-infiltrating CD8^+^ effector T cells cocultured with tumor-infiltrating TIM-1^+^ B cells or TIM-1^−^ B cells. (B) Flow cytometry analysis of the TNF-α and IFN-γ production of CD8^+^ effector T cells in the TILs and TILs without TIM-1^+^B cells groups. (TIF 269 kb)

Additional file 9: **Figure S3.** HMGB1 was expressed on the tumor-derived exosomal membrane. (A) Flow cytometry analysis of HMGB1 expression by tumor-derived exosomes and hepatocyte-derived exosomes. (TIF 101 kb)

Additional file 10: **Figure S4.** HMGB1 produced in culture was not derived from the active or passive release of HMGB1 by B cells. (A) Tumor-derived exosomes were cultured in the absence or presence of CD19+ B cells in vitro for 72 h. The supernatants from these cultures were collected, and the production of HMGB1 was determined by ELISA (n = 3). (TIF 48 kb)

Additional file 11: **Figure S5.** Tumor-derived exosomes promoted TIM-1^+^B cell expansion via TLR2/4. (A) On day 3, CD19^+^ B cells (2 × 10^5^ cells/well) purified from healthy donor PBMCs were treated with exosomes (derived from HepG2 or Huh7 cells) in the presence or absence of TLR-2 (20 μg/ml), TLR-4 (20 μg/ml), TLR-9 (75 μM) and RAGE (50 nM) inhibitors in 96-well plates and were analyzed by flow cytometry to assess the frequency of TIM-1^+^ B cells. (B) Dot plots represent the average percentages of TIM-1^+^ B cells after culturing with exosomes in the presence or absence of the TLR-2, TLR-4, TLR-9 and RAGE inhibitors (n = 3). *P < 0.05, **P < 0.01, *** *P* < 0.001. (TIF 297 kb)

Additional file 12: **Figure S6.** Tumor-derived exosomes did not promote TIM-1^+^B cell expansion via autophagy. (A) B cells were cultured with TDEs (derived from HepG2 or Huh7 cells), and the level of the autophagy-related protein LC3B in B cells was determined by western blotting. (TIF 59 kb)

Additional file 13: **Figure S7.** A schematic showing the novel mechanism of TIM-1^+^ Breg cells in HCC progression. Based on our data, we propose a model involving TIM-1^+^ Breg cells in HCC progression. First, HCC cells release exosomes with the potential to promote the accumulation of TIM-1^+^Breg cells through the HMGB1-TLR2/4-MAPK pathway. Second, TIM-1^+^Breg cells create an immunosuppressive microenvironment through secreting IL-10 and impairing CD8^+^ T cell functions, which provide favorable conditions for HCC progression. Third, myeloid cells further strengthen the immunosuppressive function of TIM-1^+^Breg cells through TIM-1/TIM-4 signaling. (TIF 13079 kb)
