# Supplementary material for: Tumor-derived exosomal HMGB1 fosters hepatocellular carcinoma immune evasion by promoting TIM-1+ regulatory B cell expansion
Source: J Immunother Cancer. 2018 Dec 10;6:145. doi: 10.1186/s40425-018-0451-6 (PMC6288912; doi:10.1186/s40425-018-0451-6)
Supplement: Supplementary file 1 — Table S1. Characteristics of the study population (N = 51. (DOCX 15 kb) [file 40425_2018_451_MOESM1_ESM.docx]

Table S1. Characteristics of the study population(N=51).

| Variable | HCC(N=51) |
| --- | --- |
| Age (years old) | 50.00±10.235  46/5  50/0/0/1  18/33  56/14/27/4  9/12/23/7  43/8  6.1±3.737  21/30  31/20 |
| Gender (Male/ Female) |  |
| HBV(+)/HCV(+)/HBV(+)HCV(+)/others  HBV-DNA(＜1*e^2^ vs ≥1*e^2^)  TNM Stage(I/II/III/IV)  Tumor Differentiation(I/II/III/IV)  Tumor Multiplicity(multilple/ solitary) |  |
| Tumor Size, cm |  |
| Tumor Microvascular invasion(No/Yes) |  |
| AFP(＜400/≥400) |  |

AFP, alpha-fetoportein; TNM, tumor, node, metastases; HBV, hepatitis B virus; HCV hepatitis C virus
