# Supplementary material for: Tumor-derived exosomal HMGB1 fosters hepatocellular carcinoma immune evasion by promoting TIM-1+ regulatory B cell expansion
Source: J Immunother Cancer. 2018 Dec 10;6:145. doi: 10.1186/s40425-018-0451-6 (PMC6288912; doi:10.1186/s40425-018-0451-6)
Supplement: Supplementary file 3 — Table S3. Flow cytometry panels for analysis of immune cells. (DOCX 17 kb) [file 40425_2018_451_MOESM3_ESM.docx]

**Table S3. Flow cytometry panels for analysis of immune cells**

| Species | Source | Antigen | Fluorochrome | Clone | Supplier |
| --- | --- | --- | --- | --- | --- |
| Human | Mouse | CD45 | BV510 | HI30 | Biolegend |
| Human | Mouse | CD19 | FITC | HIB19 | eBioscience |
| Human | Mouse | CD19 | Alexa-eFlour700 | HIB19 | Biolegend |
| Human | Mouse | CD19 | BV421 | HIB19 | Biolegend |
| Human | Mouse | CD5 | APC-Cy7 | HCHT2 | Biolegend |
| Human | Mouse | CD24 | PECY-Cy5.5 | ML5 | Biolegend |
| Human | Mouse | CD38 | PE-Cy7 | HIT2 | Biolegend |
| Human | Mouse | TIM1 | APC | 1D12 | Biolegend |
| Human | Mouse | TIM1 | Alexa-eFlour647 | 1D12 | BD Biosciences |
| Human | Mouse | TIM1 | PE | 1D12 | Biolegend |
| Human | Mouse | CD27 | PE | M-T2T1 | Biolegend |
| Human | Mouse | IL10 | BV421 | JES3-9D7 | Biolegend |
| Human | Mouse | CD8 | PE | HIT8a | Biolegend |
| Human | Mouse | TNFα | APC-Cy7 | MAb11 | Biolegend |
| Human | Mouse | IFNγ | APC | 4S.B3 | Biolegend |
| Human | Mouse | CD11b | PE | ICRF44 | eBioscience |
| Human | Mouse | CD14 | FITC | 61D3 | eBioscience |
| Human | Mouse | TIM4 | PE-Cy7 | 9F4 | Biolegend |
| Human | Mouse | CD8 | FITC | RPA-T8 | Biolegend |
| Human | Mouse | CD63 | FITC | H5C6 | BD Biosciences |
| Human | Mouse | CD81 | FITC | JS-81 | BD Biosciences |
