# Supplementary material for: Tumor-derived exosomal HMGB1 fosters hepatocellular carcinoma immune evasion by promoting TIM-1+ regulatory B cell expansion
Source: J Immunother Cancer. 2018 Dec 10;6:145. doi: 10.1186/s40425-018-0451-6 (PMC6288912; doi:10.1186/s40425-018-0451-6)
Supplement: Supplementary file 4 — Table S4. The antibodies used in our experiments. (DOCX 17 kb) [file 40425_2018_451_MOESM4_ESM.docx]

**Table S4. The antibodies used in our experiments**

| Species | Source | Antigen | Clone | Supplier | Dilution |
| --- | --- | --- | --- | --- | --- |
| Human | Rabbit | P38 | D13E1 | Cell Signaling Technology | 1:1000 |
| Human | Rabbit | ERK | 137F5 | Cell Signaling Technology | 1:1000 |
| Human | Rabbit | JNK | 56G8 | Cell Signaling Technology | 1:1000 |
| Human | Rabbit | P-P38 | D3F9 | Cell Signaling Technology | 1:1000 |
| Human | Rabbit | P-ERK | 20G11 | Cell Signaling Technology | 1:1000 |
| Human | Rabbit | P-JNK | 98F2 | Cell Signaling Technology | 1:1000 |
| Human | Rabbit | HMGB1 | N/A | NOVUS | 1:1000 |
| Human | Mouse | CD63 | TS63 | Abcam | 1:1000 |
| Human | Mouse | TSG101 | 4A10 | Abcam | 1:1000 |
| Human | Mouse | ALIX | 3A9 | Abcam | 1:1000 |
| Human | Mouse | β-tubulin | AC021 | ABclone | 1:1000 |
| Human | Chicken | antiHMGB1 | N/A | Shino-test | 1mg/ml |
| Human | Rabbit | TIM4 | N/A | NOVUS | 1:50 |
| Human | mouse | CD20 | L26 | Abcam | 1:50 |
