# Supplementary figures and images for: Tumor-derived exosomal HMGB1 fosters hepatocellular carcinoma immune evasion by promoting TIM-1+ regulatory B cell expansion
Source: J Immunother Cancer. 2018 Dec 10;6:145. doi: 10.1186/s40425-018-0451-6 (PMC6288912; doi:10.1186/s40425-018-0451-6)

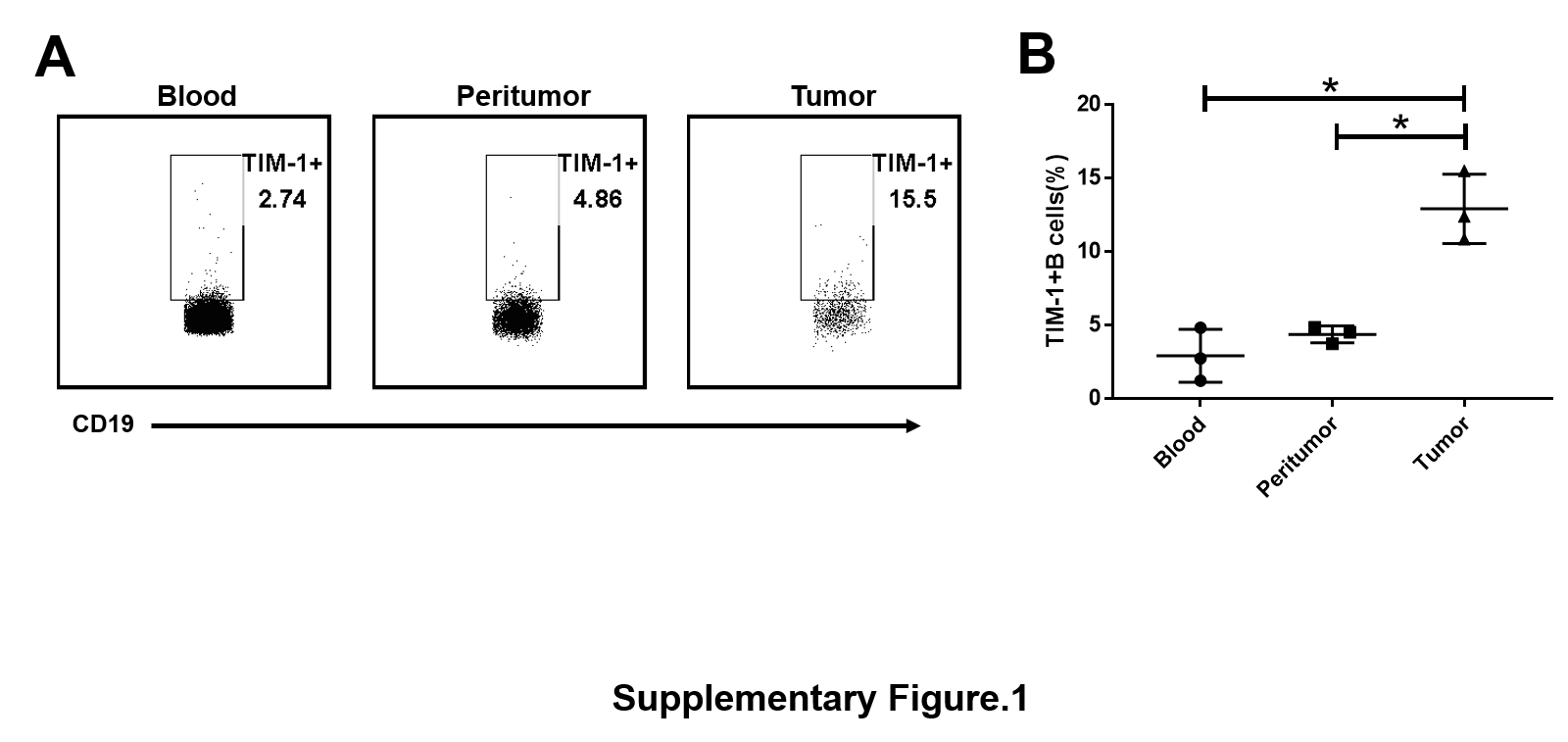

Supplement: Supplementary file 5 — Figure S1. TIM-1+ B cells strongly infiltrated lung carcinoma tissue. (A) The TIM-1+B cells in the tumor tissue were compared to those of the paired PBMC and peritumoral lung samples (n = 3). (B) The data are represented as the mean ± s.e.m. *P < 0.05. (TIF 173 kb) [file 40425_2018_451_MOESM5_ESM.tif]

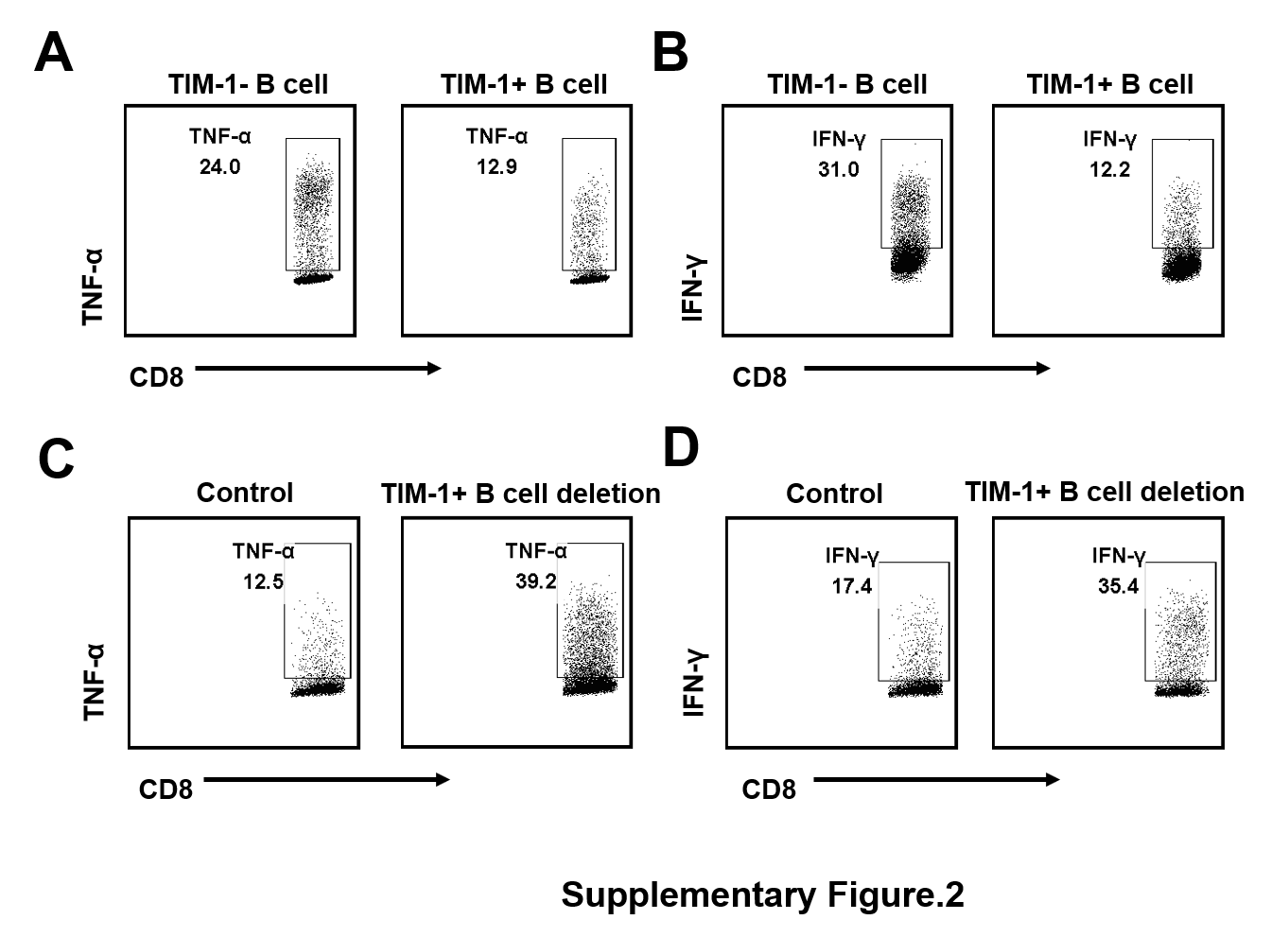

Supplement: Supplementary file 8 — Figure S2. TIM-1+Breg cells are a regulatory B cell subset that promoted HCC progression by impairing the function of CD8+ effector T cells. (A) Flow cytometry analysis of the TNF-α and IFN-γ production of tumor-infiltrating CD8+ effector T cells cocultured with tumor-infiltrating TIM-1+ B cells or TIM-1− B cells. (B) Flow cytometry analysis of the TNF-α and IFN-γ production of CD8+ effector T cells in the TILs and TILs without TIM-1+B cells groups. (TIF 269 kb) [file 40425_2018_451_MOESM8_ESM.tif]

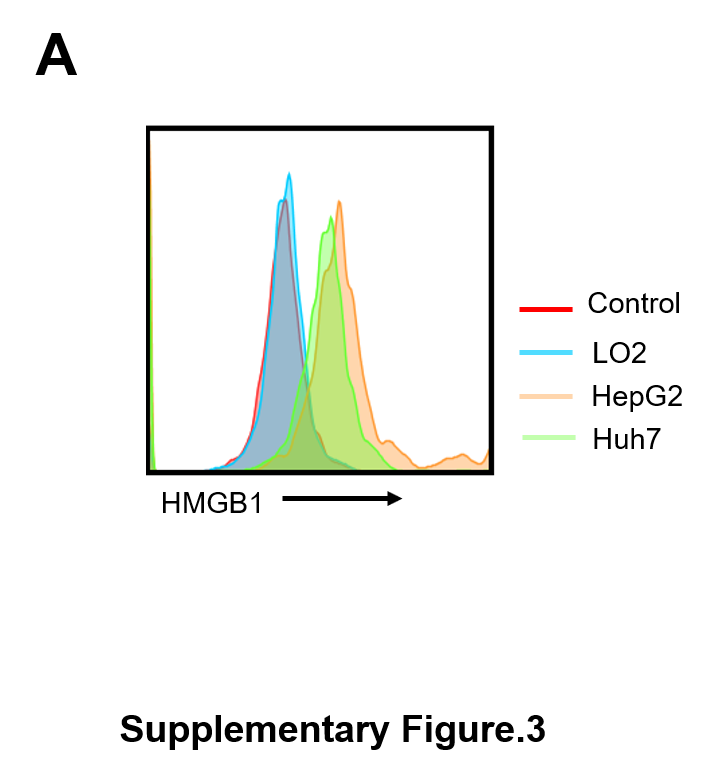

Supplement: Supplementary file 9 — Figure S3. HMGB1 was expressed on the tumor-derived exosomal membrane. (A) Flow cytometry analysis of HMGB1 expression by tumor-derived exosomes and hepatocyte-derived exosomes. (TIF 101 kb) [file 40425_2018_451_MOESM9_ESM.tif]

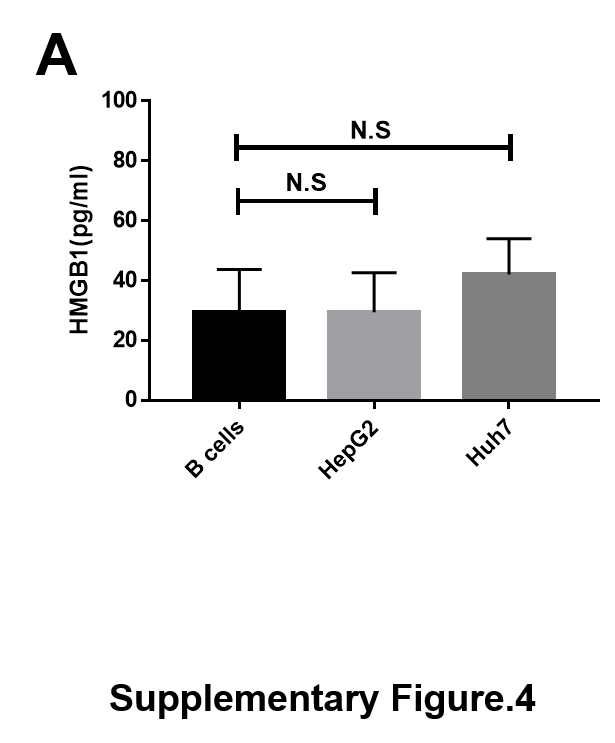

Supplement: Supplementary file 10 — Figure S4. HMGB1 produced in culture was not derived from the active or passive release of HMGB1 by B cells. (A) Tumor-derived exosomes were cultured in the absence or presence of CD19+ B cells in vitro for 72 h. The supernatants from these cultures were collected, and the production of HMGB1 was determined by ELISA (n = 3). (TIF 48 kb) [file 40425_2018_451_MOESM10_ESM.tif]

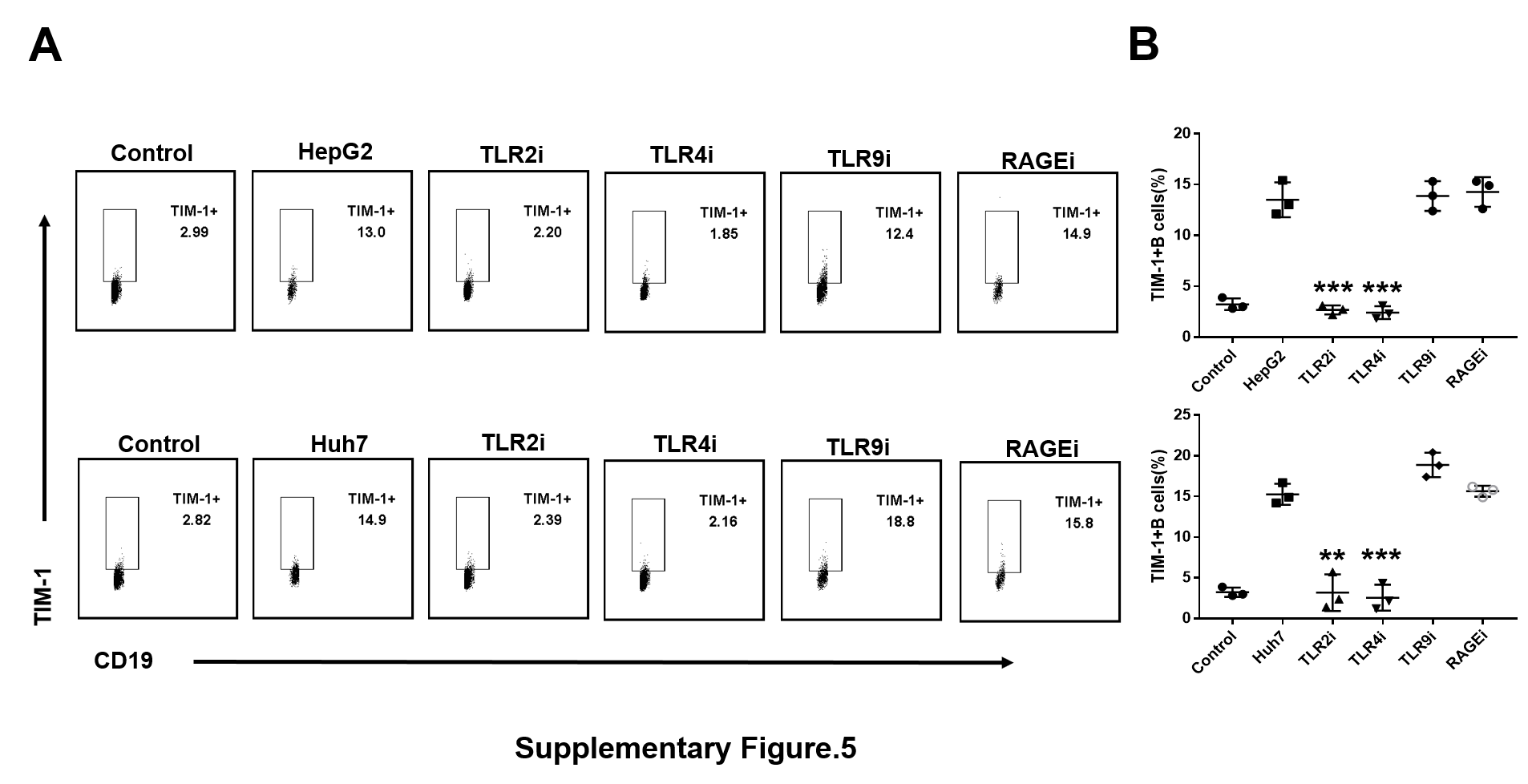

Supplement: Supplementary file 11 — Figure S5. Tumor-derived exosomes promoted TIM-1+B cell expansion via TLR2/4. (A) On day 3, CD19+ B cells (2 × 10^5^ cells/well) purified from healthy donor PBMCs were treated with exosomes (derived from HepG2 or Huh7 cells) in the presence or absence of TLR-2 (20 μg/ml), TLR-4 (20 μg/ml), TLR-9 (75 μM) and RAGE (50 nM) inhibitors in 96-well plates and were analyzed by flow cytometry to assess the frequency of TIM-1+ B cells. (B) Dot plots represent the average percentages of TIM-1+ B cells after culturing with exosomes in the presence or absence of the TLR-2, TLR-4, TLR-9 and RAGE inhibitors (n = 3). *P < 0.05, **P < 0.01, *** P < 0.001. (TIF 297 kb) [file 40425_2018_451_MOESM11_ESM.tif]

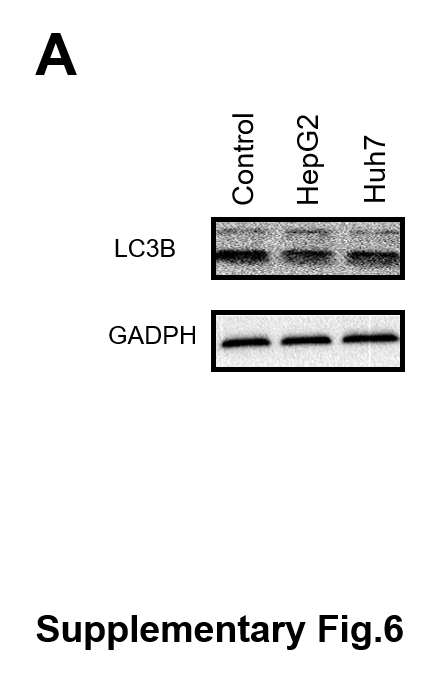

Supplement: Supplementary file 12 — Figure S6. Tumor-derived exosomes did not promote TIM-1+B cell expansion via autophagy. (A) B cells were cultured with TDEs (derived from HepG2 or Huh7 cells), and the level of the autophagy-related protein LC3B in B cells was determined by western blotting. (TIF 59 kb) [file 40425_2018_451_MOESM12_ESM.tif]

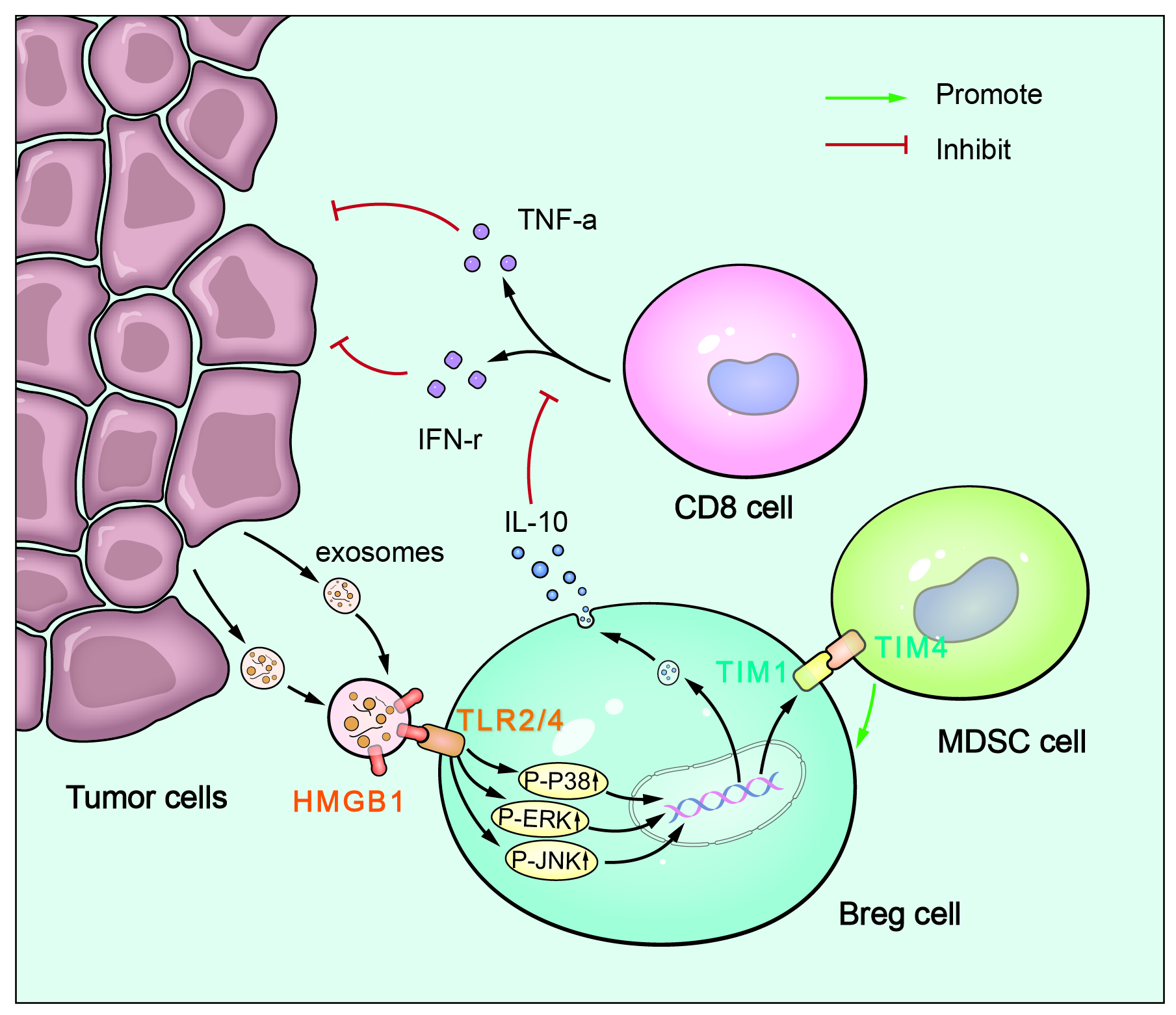

Supplement: Supplementary file 13 — Figure S7. A schematic showing the novel mechanism of TIM-1+ Breg cells in HCC progression. Based on our data, we propose a model involving TIM-1+ Breg cells in HCC progression. First, HCC cells release exosomes with the potential to promote the accumulation of TIM-1+Breg cells through the HMGB1-TLR2/4-MAPK pathway. Second, TIM-1+Breg cells create an immunosuppressive microenvironment through secreting IL-10 and impairing CD8+ T cell functions, which provide favorable conditions for HCC progression. Third, myeloid cells further strengthen the immunosuppressive function of TIM-1+Breg cells through TIM-1/TIM-4 signaling. (TIF 13079 kb) [file 40425_2018_451_MOESM13_ESM.tif]
