# Supplementary material for: Tumor-derived exosomal HMGB1 fosters hepatocellular carcinoma immune evasion by promoting TIM-1+ regulatory B cell expansion
Source: J Immunother Cancer. 2018 Dec 10;6:145. doi: 10.1186/s40425-018-0451-6 (PMC6288912; doi:10.1186/s40425-018-0451-6)
Supplement: Supplementary file 6 — Table S5. Univariate and Multivariate Analysis of Prognostic Factors for recurrence-free survival and Overall Survival (N = 29). (DOCX 18 kb) [file 40425_2018_451_MOESM6_ESM.docx]

Table S5. Univariate and Multivariate Analysis of Prognostic Factors for recurrence-free survival and Overall Survival(N=29)

| Variable | Recurrence-Free Survival | | | |
| --- | --- | --- | --- | --- |
|  | Univariate | | Multivariate | |
|  | HR  (95%CI ) | P value | HR  (95%CI ) | P value |
| Age (years old), ≤50 vs ＞50 | 1.079 (0.374-3.114) | 0.888 |  |  |
| Gender (Male vs Female) | 2.709(0.599-12.259) | 0.178 |  |  |
| Tumor Multiplicity(multilple vs solitary) | 3.013(0.808-11.235) | 0.084 |  |  |
| Tumor Size, cm(>5 vs ≤5) | 0.851(0.295-2.455) | 0.765 |  |  |
| Tumor Differentiation(III+IV vs I+II) | 0.797(0.326-1.950) | 0.620 |  |  |
| Tumor Microvascular invasion(yes vs no) | 1.406(0.470-4.201) | 0.540 |  |  |
| TNM Stage(III+IV vs I+II) | 0.796(0.516-1.227) | **0.297** |  |  |
| AFP(＜400 vs ≥400) | 1.127(0.377-3.367) | 0.831 |  |  |
| HBV-DNA(＜1*e^2^ vs ≥1*e^2^) | 1.066(0.334-3.405) | 0.914 |  |  |
| TIM1 | 3.284(1.026-10.257) | **0.034** | 0.534(0.301-0.949) | **0.033** |
|  |  |  |  |  |

AFP, alpha-fetoportein; TNM, tumor, node, metastases; HBV, hepatitis B virus; HCV hepatitis C virus
