# Supplementary material for: Tumor-derived exosomal HMGB1 fosters hepatocellular carcinoma immune evasion by promoting TIM-1+ regulatory B cell expansion
Source: J Immunother Cancer. 2018 Dec 10;6:145. doi: 10.1186/s40425-018-0451-6 (PMC6288912; doi:10.1186/s40425-018-0451-6)
Supplement: Supplementary file 7 — Table S6. Univariate and Multivariate Analysis of Prognostic Factors for recurrence-free survival and Overall Survival (N = 101). (DOCX 19 kb) [file 40425_2018_451_MOESM7_ESM.docx]

Table S6. Univariate and Multivariate Analysis of Prognostic Factors for recurrence-free survival and Overall Survival(N=101)

| Variable | Recurrence-Free Survival | | | | Overall Survival | | | |
| --- | --- | --- | --- | --- | --- | --- | --- | --- |
|  | Univariate | | Multivariate | | Univariate | | Multivariate | |
|  | HR  (95%CI ) | P value | HR  (95%CI ) | P value | HR  (95%CI ) | P value | HR  (95%CI ) | P value |
| Age (years old), ≤50 vs ＞50 | 1.124(0.646-1.958) | 0.679 |  |  | 0.989(0.502-1.948) | 0.975 |  |  |
| Gender (Male vs Female) | 1.036(0.411-2.612) | 0.941 |  |  | 1.093(0.557-2.147) | 0.795 |  |  |
| Tumor Multiplicity(multilple vs solitary) | 1.054(0.474-2.344) | 0.897 |  |  | 0.937(0.329-2.666) | 0.903 |  |  |
| Tumor Size, cm(>5 vs ≤5) | 0.823(0.467-1.450) | 0.501 |  |  | 0.482(0.246-0.946) | **0.034** | 0.389(0.194，0.779) | **0.008** |
| Tumor Differentiation(III+IV vs I+II) | 0.681(0.307-1.512) | 0.346 |  |  | 0.986(0.408-2.383) | 0.975 |  |  |
| Tumor Microvascular invasion(yes vs no) | 1.039(0.554-1.950) | 0.904 |  |  | 0.577(0.284-1.170) | 0.127 |  |  |
| TNM Stage(III+IV vs I+II) | 0.533(0.294-0.967) | **0.039** | 0.534(0.294-0.990) | **0.046** | 0.681(0.330-1.403) | 0.297 |  |  |
| AFP(＜400 vs ≥400) | 0.887(0.485-1.622) | 0.696 |  |  | 0.817(0.390-1.712) | 0.593 |  |  |
| HBV-DNA(＜1*e^2^ vs ≥1*e^2^) | 1.760(0.881-3.516) | 0.105 |  |  | 1.318(0.573-3.031) | 0.514 |  |  |
| TIM1 | 0.510(0.292-0.889) | **0.018** | 0.534(0.301-0.949) | **0.033** | 0.382(0.190-0.768) | **0.007** | 0.311  (0.151-0.642) | **0.002** |

AFP, alpha-fetoportein; TNM, tumor, node, metastases; HBV, hepatitis B virus
